# Supplementary material for: Linking structural and functional changes during aging using multilayer brain network analysis
Source: Commun Biol. 2024 Feb 28;7:239. doi: 10.1038/s42003-024-05927-x (PMC10902297; doi:10.1038/s42003-024-05927-x)
Supplement: Supplementary file 2 — Supplementary information [file 42003_2024_5927_MOESM2_ESM.pdf]

Complete characterization of the cognitive tests. These descriptions are based on what can be found on the site https://www.cam-can.org/ and in [1,2]

MMSE (Mini-Mental State Examination): Test comprising a series of 30 questions divided into 6 categories: evaluation of spatial and temporal orientation abilities, learning abilities, attention and calculation abilities, information recall abilities, language and praxis abilities.

VSTM (Visual Short-Term Memory): View (1–4) coloured discs briefly presented on a computer screen, then after a delay, attempt to remember the colour of the disc that was at a cued location, with response indicated by selecting the colour on a colour wheel (touchscreen input).

Cattell: The test contains four subtests with different types of nonverbal "puzzles": series completion, classification, matrices, and conditions. Correct responses are given a score of 1 for a total maximum score of 46.

Hotel task: Perform tasks in role of hotel manager: write customer bills, sort money, proofread advert, sort playing cards, alphabetise list of names. Total time must be allocated equally between tasks; there is not enough time to complete any one task. The number of actions performed (Hotel_num) and the time taken (Hotel_time) are measured.

Figure S1: Description of regions with statistically significant differences in multiplex participation coefficients between young and old groups

A

Regions	Participation Young group	Participation Old group	p-value	Effect	Frequency bands
**Inferiortemporal L**	0.901 (0.129)	0.790 (0.277)	≤ 0.041	**Smaller** participation in older adults	Alpha, Beta, Theta
Rostralmiddlefrontal L	0.840 (0.168)	0.726 (0.336)	≤ 0.042	**Smaller** participation in older adults	Delta, Gamma
Enthorinal L	0.686 (0.318)	0.821 (0.272)	≤ 0.031	**Bigger** participation in older adults	Alpha, Delta
**Supramarginal R**	0.668 (0.343)	0.815 (0.245)	≤ 0.029	**Bigger** participation in older adults	Alpha, Delta, Theta
Insula R	0.473 (0.383)	0.635 (0.365)	0.040	**Bigger** participation in older adults	Theta

B

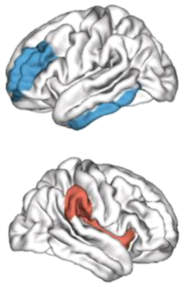

Figure S1. (**a**) Table of the five brain regions where multiplex participation coefficient was found to differ between the two groups, young and old (t-test), with the two regions in bold showing significant associations with behavioral performance. (**b**) Visualization of the five brain regions. In blue, regions where multiplex participation coefficient was decreased; in green, regions where multiplex participation coefficient was increased in the older group compared to the younger group. All results were adjusted for multiple comparisons using FDR corrections at $q < 0.05$. Values in parentheses represent deviations from the mean.

Table S1. Demographics and scores for younger group and older subgroups participants formed from multiplex participation coefficient in left temporal region.

Variables	Young adults	Older adults		p-value	t-value
		High participation	Low participation	-	-
Number of participants	46	19	19	-	-
Number of women	29	9	10	-	-
**Age**	**26.5 (2.01)**	**64.4 (2.98)**	**64.5 (2.84)**	**< 0.001**	-70.442
Years of education	22.2 (2.87)	20.1 (3.3)	19.2 (3.2)	**< 0.015**	3.659
MMSE	29.5 (0.863)	28.8 (1.4)	29 (1.05)	**0.041/**0.051	2.388/2.367
VSTM_all	0.5 (0.0875)	0.430 (0.057)	0.430 (0.081)	**<0.004**	3.985
Cattell	37.8 (3.63)	30.84 (6.89)	30.57 (6.50)	**<0.001**	6.192
Hotel_Num_rows	4.7 (0.585)	4.21 (0.91)	4.36 (1.16)	**0.009/**0.108	2.394/1.758
Hotel_Time	227.7 (120)	332.2 (181.9)	334.7 (210.3)	**< 0.013**	-3.014
**Inferiortemporal_participation PLV**	**0.902 (0.125)**	**0.977 (0.0217)**	**0.552 (0.295)**	**< 0.013**	2.792
**Inferiortemporal_participation TE**	**0.379 (0.37)**	**0.784 (0.25)**	**0.494 (0.32)**	**< 0.001/**0.244	0.485/-0.127

The p-values correspond to the highest p-value between the younger group and both older subgroups when older subgroups differ. In bold: p-value statistically different. Underlined: p-value between the younger and the older Low participation groups when older subgroups differ.

VSTM: Visual Short-Term Memory; PLV: Phase Locking Value; TE: Transfer Entropy

Table S2. Demographics and scores for both older subgroups participants formed from multiplex participation coefficient in left temporal region.

Variables	Older adults		p-value	t-value
	High participation	Low participation	**-**	**-**
Number of participants	19	19	-	-
Number of women	9	10	-	-
Age	64.4 (2.98)	64.5 (2.84)	0.960	0.545
Years of education	20.1 (3.3)	19.2 (3.2)	0.455	-0.522
MMSE	28.8 (1.4)	29 (1.05)	0.799	-0.243
VSTM_all	0.430 (0.057)	0.430 (0.081)	0.992	-0.048
Cattell	30.84 (6.89)	30.57 (6.50)	0.904	-0.447
Hotel_Num_rows	4.21 (0.91)	4.36 (1.16)	0.645	0.375
Hotel_Time	332.2 (181.9)	334.7 (210.3)	0.970	0.062
**Inferiortemporal_participation PLV**	**0.977 (0.0217)**	**0.552 (0.295)**	**<0.001**	-5.768
**Inferiortemporal_participation TE**	**0.784 (0.25)**	**0.494 (0.32)**	**0.004**	-0.771

In bold: p-value statistically different between both older subgroups.

VSTM: Visual Short-Term Memory; PLV: Phase Locking Value; TE: Transfer Entropy

Table S3. Demographics and scores for younger group and older subgroups participants formed from multiplex participation coefficient in right parietal region.

Variables	Young adults	Older adults		p-value	t-value
		High participation	Low participation	-	-
Number of participants	46	19	19	-	-
Number of women	29	8	11	-	-
**Age**	**26.5 (2.01)**	**63.913 (2.85)**	**65.087 (2.84)**	**< 0.001**	69.899
Years of education	22.2 (2.87)	19.3 (2.81)	19 (2.34)	**< 0.001**	-4.462
MMSE	29.5 (0.863)	**29.31 (0.88)**	**28.52 (1.42)**	0.440 / **0.001**	-2.541/3.338
VSTM_all	0.5 (0.0875)	0.428 (0.070)	0.443 (0.069)	**<0.014**	-3.766
Cattell	37.8 (3.63)	29.78 (5.23)	31.47 (6.89)	**<0.001**	-6.668
Hotel_Num_rows	4.7 (0.5852)	4.452 (0.69)	4.21 (1.27)	0.242 / **0.030**	-2.060/2.279
Hotel_Time	227.7 (119.7)	281.5 (144.14)	347.5 (232.05)	0.129/**0.009**	2.552/-2.763
**Supramarginal_participation PLV**	**0.668 (0.3205)**	**0.981 (0.016)**	**0.601 (0.25)**	**<0.001 /** 0.447	1.626/0.960
**Supramarginal_participation TE**	**0.351 (0.37)**	**0.634 (0.36)**	**0.341 (0.40)**	**0.007/**0.923	1.500/0.171

The p-values correspond to the highest p-value between the younger group and both older subgroups when older subgroups differ. In bold: p-value statistically different. Underlined: p-value between the younger and the older Low participation groups when older subgroups differ.

VSTM: Visual Short-Term Memory; PLV: Phase Locking Value; TE: Transfer Entropy

Table S4. Demographics and scores for both older subgroups participants formed from multiplex participation coefficient in right parietal region.

Variables	Older adults		p-value	t-value
	High participation	Low participation	-	-
Number of participants	19	19	-	-
Number of women	8	11	-	-
Age	63.913 (2.85)	65.087 (2.84)	0.244	1.349
Years of education	19.3 (2.81)	19 (2.34)	0.718	-0.363
**MMSE**	**29.31 (0.88)**	**28.52 (1.42)**	**0.048**	**-2.048**
VSTM_all	0.428 (0.070)	0.443 (0.069)	0.521	0.649
Cattell	29.78 (5.23)	31.47 (6.89)	0.402	0.848
Hotel_Num_rows	4.452 (0.69)	4.21 (1.27)	0.349	-0.949
Hotel_Time	281.5 (144.14)	347.5 (232.05)	0.300	1.052
**Supramarginal_participation PLV**	**0.981 (0.016)**	**0.601 (0.25)**	**<0.001**	**-6.428**
**Supramarginal_participation TE**	**0.634 (0.36)**	**0.341 (0.40)**	**0.023**	**-2.372**

In bold: p-value statistically different between both older subgroups.

VSTM: Visual Short-Term Memory; PLV: Phase Locking Value; TE: Transfer Entropy

Figure S2: Multiplex participation coefficient level differences between young and older subgroups and association with cognition using Phase Lag Index (wPLI)

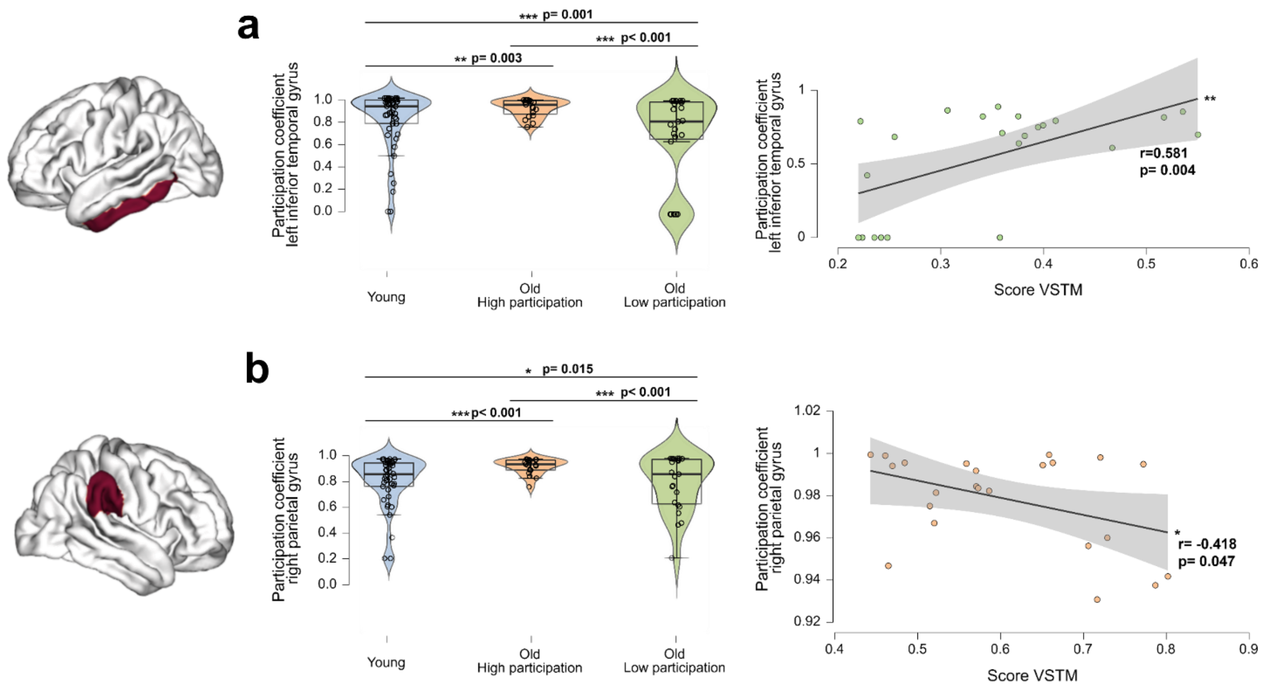

Figure S2. Multiplex participation coefficient level differences between young and older subgroups and association with cognition using Phase Lag Index (wPLI). (**a**) Distribution of young adults and older adults' subgroups for the multiplex participation coefficient in the left temporal region for the measure of synchrony (wPLI) in the alpha frequency band. Positive association between participation in the left temporal region and VSTM scores for the Low participation subgroup (regression test r= 0.581, p=0.004; no association with cognition for the High participation older subgroup). (**b**) Distribution of the young adults and older adults' subgroups for the multiplex participation coefficient in the right parietal region in alpha frequency band. Negative association between participation in the right parietal region and VSTM scores for the High participation older subgroup (regression test r= -0.418, p=0.047; no association with cognition for the Low participation older subgroup). All results were adjusted for multiple comparisons using FDR corrections at $q < 0.05$.

*p<0.05; **p<0.01; ***p<0.001

Figure S3: Changing network directed couplings in left temporal region with age is cognitively beneficial for older adults

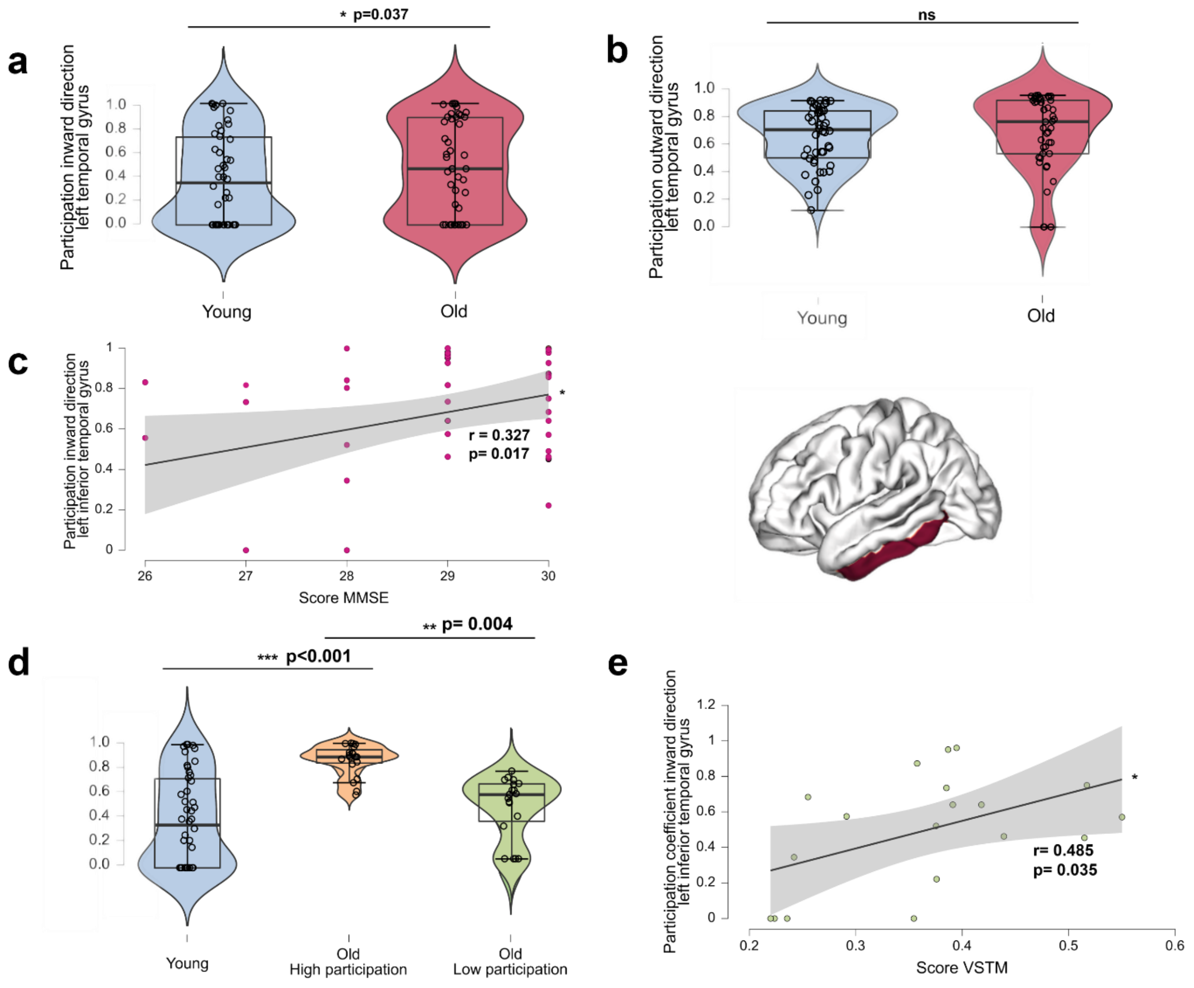

Figure S3. (**a**) Increased inward directionality (i.e., directed towards the right parietal region) in the older group compared to the younger (t-test, p=0.037) for the left temporal region in the gamma frequency band. (**b**) Preserved outward direction (i.e., directed towards other regions of the network) in the older group compared to the younger for the left temporal region, in the gamma frequency band. (**c**) Positive association between the increased of multiplex participation coefficient in inward direction in left temporal region in gamma1 frequency band and MMSE test (r= 0.327, p=0.017) in the older group. (**d**) Distribution of the young adults and older adults' subgroups for the multiplex participation coefficient in the left temporal region in gamma frequency band for the measure of directionality. (**e**) Positive association for the level of participation of left temporal region and VSTM score for the Low participation older subgroup (regression test, r= 0.485, p=0.035; no association with cognition for the High participation older subgroup). All results were adjusted for multiple comparisons using FDR corrections at $q < 0.05$

*p<0.05; **p<0.01; ***p<0.001

Figure S4: Balance of connectivity between the three layers in older group

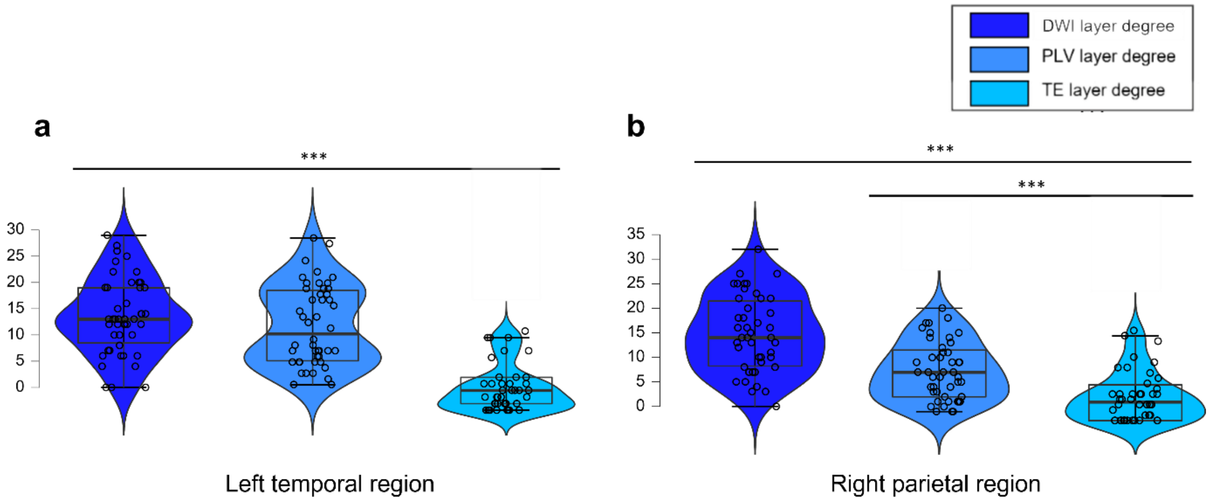

Figure S4. (**a**) Balance of connectivity in the tree layers (DWI, PLV and TE) in the left temporal region in alpha frequency band in the older group. In dark blue, the degree of the DWI layer, in midlightblue, the degree of PLV, in light blue, the degree of the TE layer. Increased of DWI degree than TE degree (p<0.001). (**b**) Balance of connectivity in the tree layers (DWI, PLV and TE) in the right parietal region in alpha frequency band in the older group. In dark blue, the degree of the DWI layer, in midlightblue, the degree of PLV, in light blue, the degree of the TE layer. Increased of DWI degree than PLV and TE degree (p<0.001).
***p<0.001

DWI: Diffusion-Weighted Imaging; PLV: Phase locking Value; TE: Transfer Entropy

Figure S5: Respective contribution of the three layers in younger group and both older subgroups

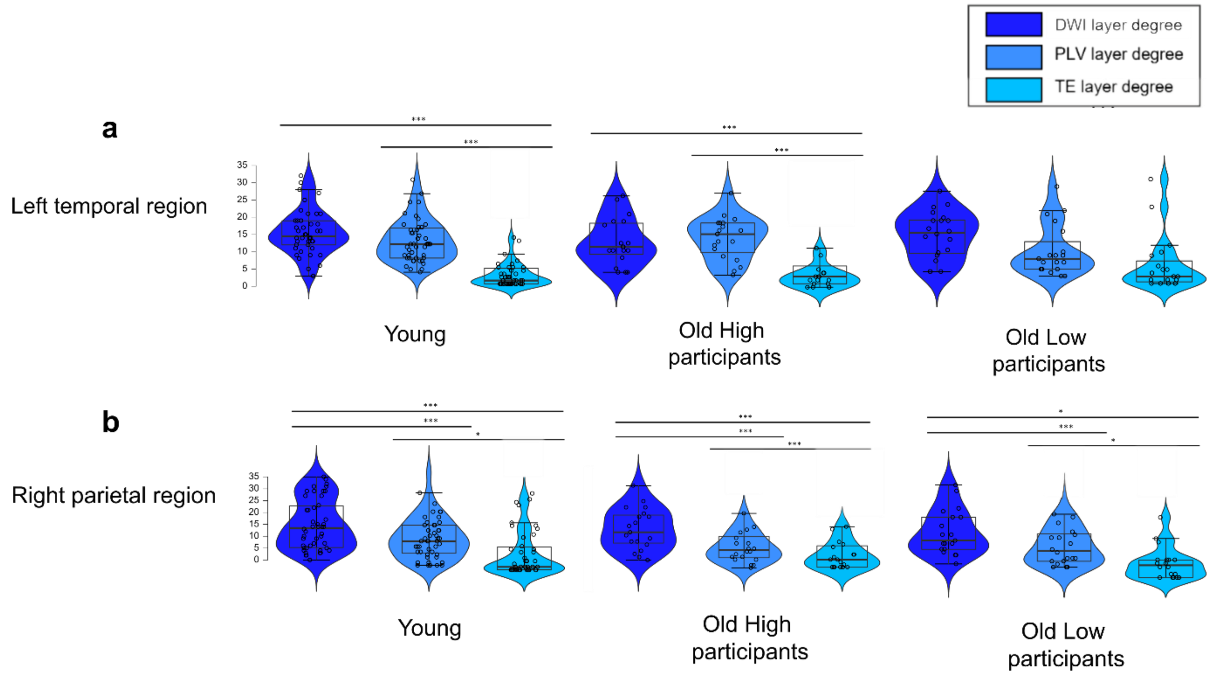

Figure S5. (**a**) Balance of connectivity in the tree layers (DWI, PLV and TE) in the left temporal region in alpha frequency band in respectively in the younger group, High participation older group and Low participation older group. In dark blue, the degree of the DWI layer, in midlightblue, the degree of PLV, in light blue, the degree of the TE layer. (**b**) Balance of connectivity in the tree layers (DWI, PLV and TE) in the right parietal region in alpha frequency band in in respectively in the younger group, High participation older group and Low participation older group. In dark blue, the degree of the DWI layer, in midlightblue, the degree of PLV, in light blue, the degree of the TE layer.

DWI: Diffusion-Weighted Imaging; PLV: Phase locking Value; TE: Transfer Entropy

Figure S6: Differences observed between young and old groups for DWI and MEG data separately

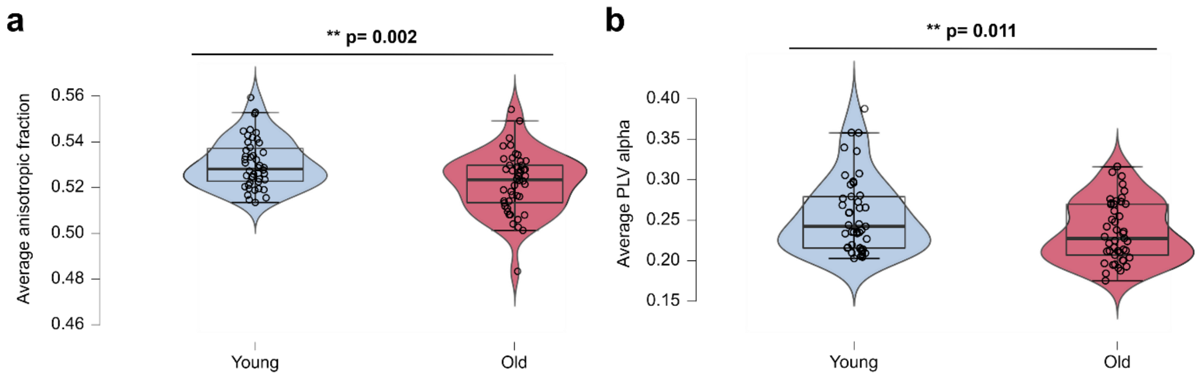

Figure S6. Differences observed between young and old groups for DWI and MEG data separately. **(a)** Greater average integrity of white matter fibers in the younger group than in the older group. **(b)** Greater average synchronization in the alpha frequency band in the younger group than in the older group.

*p<0.05

Figure S7: Flow chart detailing the participant selection process

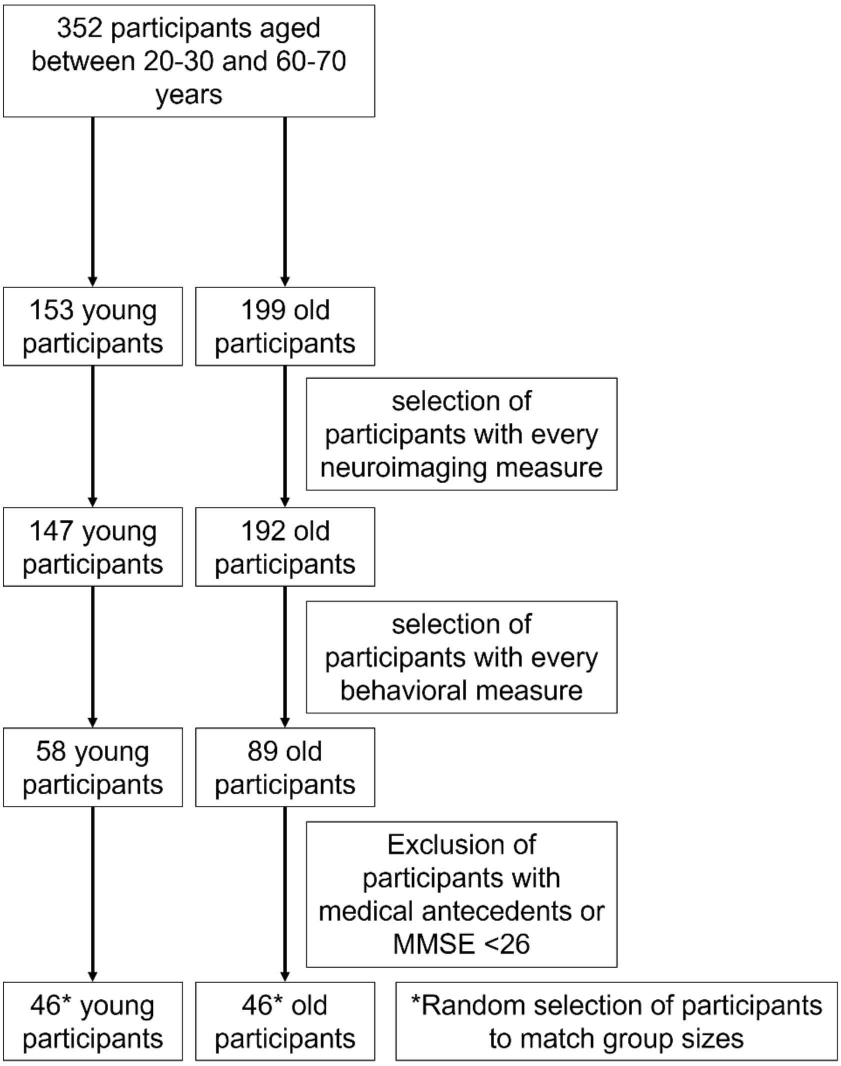

Figure S7. Flow chart detailing the participant selection process.

Table S5. Individual alpha peaks frequency (IAF) and associated frequency bands for each participant.

Participants	IAF	Delta_start	Delta_end	Theta_start	Theta_end	Alpha_start	Alpha_end	Beta_start	Beta_end	Gammas_start	Gamma_end
CC110045	9	1	3	3	7	7	11	11	23	24	89
CC110087	11	3	5	5	9	9	13	13	25	26	91
CC110098	9,5	1,5	3,5	3,5	7,5	7,5	11,5	11,5	23,5	24,5	89,5
CC110126	9	1	3	3	7	7	11	11	23	24	89
CC110174	10	2	4	4	8	8	12	12	24	25	90
CC110319	10	2	4	4	8	8	12	12	24	25	90
CC110411	10	2	4	4	8	8	12	12	24	25	90
CC112141	10	2	4	4	8	8	12	12	24	25	90
CC120065	11	3	5	5	9	9	13	13	25	26	91
CC120120	11	3	5	5	9	9	13	13	25	26	91
CC120166	11	3	5	5	9	9	13	13	25	26	91
CC120182	10	2	4	4	8	8	12	12	24	25	90
CC120208	11	3	5	5	9	9	13	13	25	26	91
CC120218	10	2	4	4	8	8	12	12	24	25	90
CC120276	12	4	6	6	10	10	14	14	26	27	92
CC120309	10	2	4	4	8	8	12	12	24	25	90
CC120313	8,5	0,5	2,5	2,5	6,5	6,5	10,5	10,5	22,5	23,5	88,5
CC120319	11	3	5	5	9	9	13	13	25	26	91
CC120469	11,5	3,5	5,5	5,5	9,5	9,5	13,5	13,5	25,5	26,5	91,5
CC120470	8	0	2	2	6	6	10	10	22	23	88
CC120727	8,5	0,5	2,5	2,5	6,5	6,5	10,5	10,5	22,5	23,5	88,5
CC120764	9	1	3	3	7	7	11	11	23	24	89
CC120795	10	2	4	4	8	8	12	12	24	25	90
CC121106	11	3	5	5	9	9	13	13	25	26	91
CC121144	11	3	5	5	9	9	13	13	25	26	91
CC121158	11,5	3,5	5,5	5,5	9,5	9,5	13,5	13,5	25,5	26,5	91,5
CC121200	10,5	2,5	4,5	4,5	8,5	8,5	12,5	12,5	24,5	25,5	90,5
CC121317	12	4	6	6	10	10	14	14	26	27	92
CC121397	8,5	0,5	2,5	2,5	6,5	6,5	10,5	10,5	22,5	23,5	88,5
CC121411	10,5	2,5	4,5	4,5	8,5	8,5	12,5	12,5	24,5	25,5	90,5
CC121428	11	3	5	5	9	9	13	13	25	26	91
CC121479	11	3	5	5	9	9	13	13	25	26	91

CC121795	8	0	2	2	6	6	10	10	22	23	88
CC122172	11	3	5	5	9	9	13	13	25	26	91
CC122405	10	2	4	4	8	8	12	12	24	25	90
CC122620	10	2	4	4	8	8	12	12	24	25	90
CC210250	9,5	1,5	3,5	3,5	7,5	7,5	11,5	11,5	23,5	24,5	89,5
CC210519	10	2	4	4	8	8	12	12	24	25	90
CC212153	10	2	4	4	8	8	12	12	24	25	90
CC220115	10	2	4	4	8	8	12	12	24	25	90
CC220352	10	2	4	4	8	8	12	12	24	25	90
CC220519	10	2	4	4	8	8	12	12	24	25	90
CC220526	7	-1	1	1	5	5	9	9	21	22	87
CC221033	10	2	4	4	8	8	12	12	24	25	90
CC221209	11	3	5	5	9	9	13	13	25	26	91
CC221373	10	2	4	4	8	8	12	12	24	25	90
CC222555	9	1	3	3	7	7	11	11	23	24	89
CC510086	10	2	4	4	8	8	12	12	24	25	90
CC510163	8	0	2	2	6	6	10	10	22	23	88
CC510258	8,5	0,5	2,5	2,5	6,5	6,5	10,5	10,5	22,5	23,5	88,5
CC510342	7,5	-0,5	1,5	1,5	5,5	5,5	9,5	9,5	21,5	22,5	87,5
CC510355	12	4	6	6	10	10	14	14	26	27	92
CC510392	8,5	0,5	2,5	2,5	6,5	6,5	10,5	10,5	22,5	23,5	88,5
CC510395	9	1	3	3	7	7	11	11	23	24	89
CC510415	8	0	2	2	6	6	10	10	22	23	88
CC510433	9	1	3	3	7	7	11	11	23	24	89
CC510486	10	2	4	4	8	8	12	12	24	25	90
CC510548	9	1	3	3	7	7	11	11	23	24	89
CC510551	9	1	3	3	7	7	11	11	23	24	89
CC510648	9	1	3	3	7	7	11	11	23	24	89
CC520002	9	1	3	3	7	7	11	11	23	24	89
CC520011	6	-2	0	0	4	4	8	8	20	21	86
CC520053	9	1	3	3	7	7	11	11	23	24	89
CC520065	8,5	0,5	2,5	2,5	6,5	6,5	10,5	10,5	22,5	23,5	88,5
CC520078	8,5	0,5	2,5	2,5	6,5	6,5	10,5	10,5	22,5	23,5	88,5

CC520097	9	1	3	3	7	7	11	11	23	24	89
CC520134	9	1	3	3	7	7	11	11	23	24	89
CC520136	9	1	3	3	7	7	11	11	23	24	89
CC520147	9	1	3	3	7	7	11	11	23	24	89
CC520200	10	2	4	4	8	8	12	12	24	25	90
CC520215	10	2	4	4	8	8	12	12	24	25	90
CC520239	9	1	3	3	7	7	11	11	23	24	89
CC520254	10	2	4	4	8	8	12	12	24	25	90
CC520279	10	2	4	4	8	8	12	12	24	25	90
CC520377	10	2	4	4	8	8	12	12	24	25	90
CC520390	10	2	4	4	8	8	12	12	24	25	90
CC520391	10	2	4	4	8	8	12	12	24	25	90
CC520395	9	1	3	3	7	7	11	11	23	24	89
CC520424	9	1	3	3	7	7	11	11	23	24	89
CC520503	10	2	4	4	8	8	12	12	24	25	90
CC520552	9	1	3	3	7	7	11	11	23	24	89
CC520585	8	0	2	2	6	6	10	10	22	23	88
CC520607	9	1	3	3	7	7	11	11	23	24	89
CC520624	10	2	4	4	8	8	12	12	24	25	90
CC520745	8,5	0,5	2,5	2,5	6,5	6,5	10,5	10,5	22,5	23,5	88,5
CC520775	10	2	4	4	8	8	12	12	24	25	90
CC520868	8,5	0,5	2,5	2,5	6,5	6,5	10,5	10,5	22,5	23,5	88,5
CC610051	9	1	3	3	7	7	11	11	23	24	89
CC610071	9,5	1,5	3,5	3,5	7,5	7,5	11,5	11,5	23,5	24,5	89,5
CC620073	10	2	4	4	8	8	12	12	24	25	90
CC620259	10	2	4	4	8	8	12	12	24	25	90
CC620262	9,5	1,5	3,5	3,5	7,5	7,5	11,5	11,5	23,5	24,5	89,5
CC620479	9,5	1,5	3,5	3,5	7,5	7,5	11,5	11,5	23,5	24,5	89,5
CC621184	10	2	4	4	8	8	12	12	24	25	90

Table S1. Individual alpha peaks frequency (IAF) and associated frequency bands for each participant.

References

1. Shafto, M. A. *et al.* The Cambridge Centre for Ageing and Neuroscience (Cam-CAN) study protocol: a cross-sectional, lifespan, multidisciplinary examination of healthy cognitive ageing. *BMC Neurology* **14**, (2014).
2. Taylor, J. R. *et al.* The Cambridge Centre for Ageing and Neuroscience (Cam-CAN) data repository: Structural and functional MRI, MEG, and cognitive data from a cross-sectional adult lifespan sample. *NeuroImage* **144**, 262–269 (2017).
