# Supplementary material for: Linking structural and functional changes during aging using multilayer brain network analysis
Source: Commun Biol. 2024 Feb 28;7:239. doi: 10.1038/s42003-024-05927-x (PMC10902297; doi:10.1038/s42003-024-05927-x)
Supplement: Supplementary file 3 — Description of Additional Supplementary Files [file 42003_2024_5927_MOESM3_ESM.pdf]

Description of Additional Supplementary Files

File name: Supplementary Data
Description: The source data behind the graphs and charts in the paper.
