# Supplementary material for: Linking structural and functional changes during aging using multilayer brain network analysis
Source: Commun Biol. 2024 Feb 28;7:239. doi: 10.1038/s42003-024-05927-x (PMC10902297; doi:10.1038/s42003-024-05927-x)
Supplement: Supplementary file 5 — Reporting Summary [file 42003_2024_5927_MOESM5_ESM.pdf]

nature portfolio

Corresponding author(s): Thomas Hinault

Last updated by author(s): Feb 15, 2024

Reporting Summary

Nature Portfolio wishes to improve the reproducibility of the work that we publish. This form provides structure for consistency and transparency in reporting. For further information on Nature Portfolio policies, see our Editorial Policies and the Editorial Policy Checklist.

Statistics

For all statistical analyses, confirm that the following items are present in the figure legend, table legend, main text, or Methods section.

n/a	Confirmed	
☐	☒	The exact sample size (*n*) for each experimental group/condition, given as a discrete number and unit of measurement
☐	☒	A statement on whether measurements were taken from distinct samples or whether the same sample was measured repeatedly
☐	☒	The statistical test(s) used AND whether they are one- or two-sided<br>*Only common tests should be described solely by name; describe more complex techniques in the Methods section.*
☐	☒	A description of all covariates tested
☐	☒	A description of any assumptions or corrections, such as tests of normality and adjustment for multiple comparisons
☐	☒	A full description of the statistical parameters including central tendency (e.g. means) or other basic estimates (e.g. regression coefficient) AND variation (e.g. standard deviation) or associated estimates of uncertainty (e.g. confidence intervals)
☐	☒	For null hypothesis testing, the test statistic (e.g. *F*, *t*, *r*) with confidence intervals, effect sizes, degrees of freedom and *P* value noted<br>*Give P values as exact values whenever suitable.*
☒	☐	For Bayesian analysis, information on the choice of priors and Markov chain Monte Carlo settings
☒	☐	For hierarchical and complex designs, identification of the appropriate level for tests and full reporting of outcomes
☒	☐	Estimates of effect sizes (e.g. Cohen's *d*, Pearson's *r*), indicating how they were calculated

Our web collection on statistics for biologists contains articles on many of the points above.

Software and code

Policy information about availability of computer code

Data collection	Data collection and sharing for this project was provided by the Cambridge Centre for Ageing and Neuroscience (CamCAN). No software was used in data collection.
Data analysis	Data were analysed using the Jamovi software (https://www.jamovi.org/; version 1.6.23).<br>Prepocessing of DTI data were performed with ExploreDTI (http://www.ExploreDTI.com). Preprocessing of MEG data were performed with the Brainstorm (http://neuroimage.usc.edu/brainstorm) toolbox implemented in Matlab (MATLAB Version: 9.13.0 (R2022b) Update 2).

For manuscripts utilizing custom algorithms or software that are central to the research but not yet described in published literature, software must be made available to editors and reviewers. We strongly encourage code deposition in a community repository (e.g. GitHub). See the Nature Portfolio guidelines for submitting code & software for further information.

Data

Policy information about availability of data

All manuscripts must include a data availability statement. This statement should provide the following information, where applicable:

- Accession codes, unique identifiers, or web links for publicly available datasets
- A description of any restrictions on data availability
- For clinical datasets or third party data, please ensure that the statement adheres to our policy

Data used in the in this study were obtained from the CamCAN repository (available at http://www.mrc-cbu.cam.ac.uk/datasets/camcan/).

Human research participants

Policy information about studies involving human research participants and Sex and Gender in Research.

Reporting on sex and gender	Sex and gender was not considered in this study.
Population characteristics	46 young healthy participants (29 females and 17 males, mean age = 26.5 years, SD = 2.01) and 46 healthy older participants (29 females and 17 males, mean age = 64.5 years, SD = 2.85). For more informations of groups characteristics see Table 1s to Table 4s of the supplementary data.
Recruitment	Data used in the in this study were obtained from the CamCAN repository.
Ethics oversight	The study was approved by the local ethics Committee: Cambridgeshire 2 Research Ethics Committee (reference: 10/H0308/50).

Note that full information on the approval of the study protocol must also be provided in the manuscript.

Field-specific reporting

Please select the one below that is the best fit for your research. If you are not sure, read the appropriate sections before making your selection.

☒ Life sciences ☐ Behavioural & social sciences ☐ Ecological, evolutionary & environmental sciences

For a reference copy of the document with all sections, see nature.com/documents/nr-reporting-summary-flat.pdf

Life sciences study design

All studies must disclose on these points even when the disclosure is negative.

Sample size	No statistical methods were used to pre-determine the sample size. But we used the largest sample of neuroimaging data reported to conduct the study.
Data exclusions	Exclusion criteria for CamCAN datatset was determined by collecting site and are listed in Shafto et al., 2014.
Replication	The reproductibility of findings is possible due to the open sources CamCAN dataset.
Randomization	This study includes two experimental groups: young and old healthy individuals. These participants were randomly included from the CamCAN database.
Blinding	Blinding was not possible and not applicable for establishing growth trajectories. All analyses were conducted in a data driven manner.

Reporting for specific materials, systems and methods

We require information from authors about some types of materials, experimental systems and methods used in many studies. Here, indicate whether each material, system or method listed is relevant to your study. If you are not sure if a list item applies to your research, read the appropriate section before selecting a response.

Materials & experimental systems

n/a	Involved in the study	
☒	☐	Antibodies
☒	☐	Eukaryotic cell lines
☒	☐	Palaeontology and archaeology
☒	☐	Animals and other organisms
☒	☐	Clinical data
☒	☐	Dual use research of concern

Methods

n/a	Involved in the study	
☒	☐	ChIP-seq
☒	☐	Flow cytometry
☐	☒	MRI-based neuroimaging

Magnetic resonance imaging

Experimental design

Design type	Resting-state

Design specifications | No specific experimental setup was used

Behavioral performance measures | No behavioural measures are included

Acquisition

Imaging type(s) | Diffusion

Field strength | 3T

Sequence & imaging parameters | DTI data were obtained with the following parameters: repetition time = 9100 ms; echo time = 104 ms; inversion time = 900 ms; field of view = 192 mm x 192 mm; 66 axial slices; voxel size = 2 mm isotropic; B0 = 0.1000/2000s/mm2; acquisition time = 10 minutes and 2 seconds, readout time 0.0684 (echo spacing = 0.72ms, EPI factor = 96). See https://camcan-archive.mrc-cbu.cam.ac.uk/dataaccess/ for more information.

Area of acquisition | Whole brain

Diffusion MRI ☒ Used ☐ Not used

Parameters | 30 diffusion gradient directions, b-value = 1000 s/mm2

Preprocessing

Preprocessing software | Prepocessing of DTI data were performed with ExploreDTI (http://www.ExploreDTI.com). It included the following steps: (a) images were corrected for eddy current distortions and participant motion; (b) a non-linear least squares method was applied for diffusion tensor estimation, and (c) deterministic DTI tractography was applied using the following parameters: uniform resolution of 2 mm, fractional anisotropy (FA) threshold of 0.2 (limit: 1), angle threshold of 45°, and fibre length range of 50 to 500 mm.

Normalization | *If data were normalized/standardized, describe the approach(es): specify linear or non-linear and define image types used for transformation OR indicate that data were not normalized and explain rationale for lack of normalization.*

Normalization template | MNI

Noise and artifact removal | *Describe your procedure(s) for artifact and structured noise removal, specifying motion parameters, tissue signals and physiological signals (heart rate, respiration).*

Volume censoring | We did not applied volume censoring

Statistical modeling & inference

Model type and settings | *Specify type (mass univariate, multivariate, RSA, predictive, etc.) and describe essential details of the model at the first and second levels (e.g. fixed, random or mixed effects; drift or auto-correlation).*

Effect(s) tested | *Define precise effect in terms of the task or stimulus conditions instead of psychological concepts and indicate whether ANOVA or factorial designs were used.*

Specify type of analysis: ☐ Whole brain ☒ ROI-based ☐ Both

Anatomical location(s) | The 68 regions of the Desikan-Killiany atlas were used as ROI.

Statistic type for inference (See Eklund et al. 2016) | *Specify voxel-wise or cluster-wise and report all relevant parameters for cluster-wise methods.*

Correction | The expected proportion of type 1 error accros multiple testing was controlled using the False Discovery Rate (FDR) correction, with a desired FDR q= .05 and assuming a positive dependency beteween conditions.

Models & analysis

n/a	Involved in the study
☐	☒ Functional and/or effective connectivity
☐	☒ Graph analysis
☒	☐ Multivariate modeling or predictive analysis

Functional and/or effective connectivity | *Report the measures of dependence used and the model details (e.g. Pearson correlation, partial correlation, mutual information).*

Graph analysis | We used binarized graphs. The measure of multiplex participation coefficient was used.
